# The effectiveness, suitability, and sustainability of non-pharmacological methods of managing pain in community-dwelling older adults: a systematic review

**DOI:** 10.1186/s12889-019-7831-9

**Published:** 2019-11-08

**Authors:** Shuk Kwan Tang, Mimi Mun Yee Tse, Sau Fong Leung, Theofanis Fotis

**Affiliations:** 1 School of Nursing, The Hong Kong Polytechnic University, Kowloon, Hong Kong; 2 School of Health Sciences, University of Brighton, Westlain House, Village Way, Brighton, BN1 9PH UK

**Keywords:** Chronic pain, Aged, Older adults, Community-dwelling, Non-pharmacological interventions, Complementary therapy, Systematic review

## Abstract

**Background:**

Pain is common in older adults. To maintain their quality of life and promote healthy ageing in the community, it is important to lower their pain levels. Pharmacological pain management has been shown to be effective in older adults. However, as drugs can have various side effects, non-pharmacological pain management is preferred for community-dwelling older adults. This systematic review evaluates the effectiveness, suitability, and sustainability of non-pharmacological pain management interventions for community-dwelling older adults.

**Methods:**

Five databases, namely, CINHAL, Journals@Ovid, Medline, PsycInfo, and PubMed, were searched for articles. The criteria for inclusion were: full-text articles published in English from 2005 to February 2019 on randomized controlled trials, with chronic non-cancer pain as the primary outcome, in which pain was rated by intensity, using non-pharmacological interventions, and with participants over 65 years old, community-dwelling, and mentally competent. A quality appraisal using the Jadad Scale was conducted on the included articles.

**Results:**

Ten articles were included. The mean age of the older adults was from 66.75 to 76. The interventions covered were acupressure, acupuncture, guided imagery, qigong, periosteal stimulation, and Tai Chi. The pain intensities of the participants decreased after the implementation of the intervention. The net changes in pain intensity ranged from − 3.13 to − 0.65 on a zero to ten numeric rating scale, in which zero indicates no pain and ten represents the worst pain.

**Conclusions:**

Non-pharmacological methods of managing pain were effective in lowering pain levels in community-dwelling older adults, and can be promoted widely in the community.

## Background

Pain is a common occurrence in humans, especially in those suffering from chronic illnesses. Trauma, injury, and illnesses can cause pain in older adults. According to the International Association for the Study of Pain, pain is ‘an unpleasant and emotional experience associated with actual or potential tissue damage or described in terms of such damage’ [1]. The prevalence of pain in community-dwelling older adults ranges from 25 to 50% [2, 3]. Persistent pain in a person for 3 months or more is recognized as chronic pain [4]. Pain originates from the musculoskeletal system [5–8], and the most common sites of pain for older adults are the back, arms, hips, and legs [5].

The American Geriatric Society provides clinical practice guidelines and recommendations on how to manage persistent pain in older persons [9]. Pain management in older adults can be complex because of the interactions between diseases. The use of analgesics is the most common method of relieving pain in older adults because of its effectiveness. When analgesics are prescribed and adjusted, special consideration and warnings have to be given to older persons who might be susceptible to the adverse effects of the analgesics [9, 10]. The following are some common adverse effects of analgesics. Gastrointestinal bleeding, oliguria, fluid retention, decreased excretion of sodium, renal failure, and prolonged bleeding can result from the use of non-steroidal anti-inflammatory drugs. Delirium, constipation, nausea, pupil constriction, and respiratory distress are the most common adverse effects brought about by morphine [11, 12]. In addition, changes in body composition, advanced age, and comorbidities can affect the pharmacokinetics and pharmacodynamics of the analgesics. The physiological changes related to ageing can affect an individual’s absorption, excretion, and response to the analgesics. The pain reduction effect can be less than expected [13].

To lower the chances of developing adverse effects from the analgesics and to improve pain control in older adults, it has been suggested that non-pharmacological interventions be used in combination with analgesics [9, 14]. Non-pharmacological interventions for pain management are ‘a number of physical and psychologic treatment modalities that often require active participation’ [9]. Examples of non-pharmacological interventions include exercise programmes and education programmes for patients and their caregivers [9, 14]. Such interventions can build self-reliance and a sense of control over pain, and it is suggested that they are ‘an integral part of the approach to the management of any persistent pain problem’ [9]. Older adults accept pain as part of the ageing process and tend not to use medication to reduce pain levels [9, 15]. Non-pharmacological interventions can thus be an alternative choice for older adults who fear the side effects of analgesics.

A pain assessment is simple to perform and is a reliable way of obtaining information about the pain condition of older adults, especially when the older adults self-report their pain condition [16, 17]. A pain assessment is ‘a fundamental process’ that should be conducted before and after an intervention [18]. Reducing the intensity of a person’s pain can be a way of measuring the effect of the intervention on pain levels. It is also essential to evaluate the suitability and sustainability of an intervention. Suitability refers to ‘the quality of being right or appropriate for a particular person, purpose or situation’ [19]. It is often assessed by obtaining the perceptions and views of the targeted population on the interventions or assessment tools [20–22]. Sustainability is ‘the ability to be maintained at a certain rate or level’ [23]. It ‘requires its own evaluation, apart from and usually after, an evaluation has shown positive results for the programme intervention itself’ [24]. Sustainability refers not only to the effects of the interventions, but also to whether organizations continue to implement the interventions even after the end of the study period. However, there is no consensus on how sustainability should be defined or measured.

Reviews of the literature on the effectiveness of non-pharmacological interventions for managing pain in older adults have previously been conducted. The authors of these reviews have presented a general picture of how pain in older adults can be managed using appropriate pain assessments, analgesics, and non-pharmacological interventions [25–30]. However, the studies that were included in these reviews were not specifically targeted at community-dwelling older adults [26, 29, 30]. Thus, the reviews lack sufficient information to evaluate and compare the effectiveness of the interventions in reducing pain intensity [25, 29]. Also, they did not discuss the suitability and sustainability of the interventions. The literature reviews also included quasi-experimental studies, pilot studies, case studies, doctoral theses, and unpublished articles [26–29]. Some literature reviews were published from the 1930s to the 2010s, and therefore could not reflect recent innovations, trends, and up-to-date information on pain management for older adults [27, 29, 30]. An appraisal of the quality of the included articles was not performed, and there may also have been some bias in the selection of these articles. Thus, it is difficult to determine the quality of these literature reviews [25–27, 29]. The authors of the reviews did not adopt guidelines for conducting the literature reviews and presenting the findings [25–30]. Thus, for the present systematic review of non-pharmacological methods of managing pain in community-dwelling older adults, it was essential to identify and appraise the current relevant literature and to provide evidence on the quality of the care delivered to the community-dwelling older adults, as well as to fill in the gaps identified in the previous literature reviews.

This systematic review included articles from 2005 to February 2019, and adopted the Preferred Reporting Items for Systematic Reviews and Meta-Analyses (PRISMA) format to guide the process of searching for articles and writing the report. It is comprised of a 27-item checklist and a four-phrase flow diagram to ‘help authors report a wide array of systematic reviews to assess the benefits and harms of a healthcare intervention’ [31, 32]. PRISMA can help to ensure that systematic reviews and meta-analyses are reported in a transparent and complete manner, and can guide authors in assessing the strengths and weaknesses of interventions in a systematic review [31]. The present systematic review includes recently published and up-to-date studies on non-pharmacological interventions for managing pain in community-dwelling older adults. Since the participants of the studies cannot be reached, the suitability and sustainability of the interventions were assessed by whether the interventions could be carried out by the participants themselves and whether they succeeded in reducing the intensity of their pain.

The research questions were as follows.

1. Are non-pharmacological interventions for managing pain effective at reducing pain intensities in community-dwelling older adults with chronic pain?
2. Are non-pharmacological interventions for pain management suitable for use in community-dwelling older adults with chronic pain?
3. Are non-pharmacological interventions for pain management sustainable in follow-up assessments of community-dwelling older adults with chronic pain?

Therefore, the objective of the present systematic review was to evaluate the effectiveness, suitability, and sustainability of non-pharmacological interventions for managing pain in community-dwelling older adults.

## Methods

### Search terms

To find articles of interest, we identified and modified search terms in consultation with a university faculty librarian with knowledge of the subject area. The following search terms were adopted: ((Pain OR Pain management)) AND (Non-pharmacological interventions) AND ((Old OR Older OR Older people OR Elderly)), (Complementary therapy), ((Non-malignant pain OR Pain management)), and (Community).

### Eligibility criteria

The following inclusion criteria were set for the systematic review.

1. Research studies published from 1 January 2005 to 28 February 2019;
2. Randomized controlled trials;
3. Articles written in English;
4. Trials assessing non-malignant chronic pain as the primary outcome;
5. Trials rating pain by intensity;
6. A non-pharmacological intervention was the sole intervention;
7. The recruited participants were community-dwelling older adults, who were not institutionalized or staying in a nursing home;
8. A criterion for being a participant in the study was being over 65 years of age;
9. The recruited participants did not suffer from any psychiatric illnesses that could affect their understanding of the interventions; and
10. A full text of the article was available.

The following works were excluded from the systematic review.

1. Book reviews;
2. Dissertations;
3. Literature reviews;
4. Study protocols;
5. Pilot studies;
6. Articles examining the effectiveness of commercial products; and
7. Articles examining the intake of traditional Chinese medications.

### Information sources

Five internet-based databases were selected for the literature search: CINHAL, Journals@Ovid, Medline, PsycInfo, and PubMed. These five databases contain medical and nursing journal articles related to pain and pain management. The literature search was conducted in March 2019.

Additional searches using Medical Subject Headings (MeSH) were performed. Medline and PubMed were searched. MeSH terms used in the additional search were chronic pain, complementary therapies, aged, and aged, 80 and over. The search terms were modified in the additional search as MeSH has developed its terminology for searching for information.

### Search and study selection

After we performed the initial search using the search terms in the five databases and the additional search of MeSH terms in the two databases, we used EndNoteX8 to remove duplicate articles [33]. The articles were then screened by title and abstract to find relevant studies on improving pain conditions and randomized controlled trials. Three independent reviewers who are experienced in conducting pain research undertook the process of screening for relevant articles. A further selection of the remaining articles was achieved by applying the inclusion and exclusion criteria to screen the abstracts and full text of the articles.

### Data collection process and data items

We designed a specific data extraction form for this systematic review. Data were extracted from the selected articles, and were reviewed by three people. Disagreements on whether certain articles should or should not be included were resolved by discussion among the three reviewers until a consensus was reached. The data extraction form contained items covering the title of the article, authors, journal, issue, year of publication, and study characteristics, including the aims of the study, study design, study duration, intervention, follow-up assessment, and recruitment procedure. The participants’ characteristics were also extracted, including the number of recruited participants, their mean age, gender, and ethnicity. Primary and secondary outcome measures were also recorded, along with the relevant assessment tools, the main findings of the studies, and the limitations.

### Suitability and sustainability of the non-pharmacological interventions

To examine the suitability and sustainability of the non-pharmacological interventions, we focused on the personnel required to deliver the interventions and the pain reduction effect. First, we looked at whether the interventions could be carried out by the participants individually or whether they had to be delivered by a therapist, healthcare professional, or a third person. Second, the sustained pain reduction effects of the interventions were explored by looking at the level of pain intensity in the follow-up assessment. The above information was noted down on the data extraction form for analysis.

### Quality appraisal

A quality appraisal was conducted using the Jadad Scale, which was developed to assess the quality of the articles in a systematic review by avoiding selection bias and examining the effect of blinding in randomized controlled trials [34]. There are five criteria in the Jadad Scale, including randomization (randomized study design and appropriateness of randomization), blinding (double-blind and appropriateness of and blinding method, and description of withdrawals and dropouts were described. Each criterion is assigned a score of one point, for a total score of five. A higher score indicates a higher quality study. In this review, an interrater consistency of 0.66 in the Jadad Scale was found [34]. Disagreements were discussed among the three reviewers and resolved.

### Summary measures

The primary outcome measure of the systematic review was a reduction in the mean intensity of pain in community-dwelling older adults after the use of non-pharmacological interventions in the studies. The mean intensity had to be presented in a numerical format, regardless of which assessment tools were adopted. The pre-intervention mean pain intensity was compared to the post-intervention mean pain intensity to determine whether there had been any improvement. Different pain assessment tools were used in the included articles. To make comparisons about the pain intensity in different studies, pain intensity levels were converted to a numeric rating scale ranging from zero to ten, with zero indicating no pain and ten representing the worst pain.

## Results

### Study selection

**Fig. 1:**
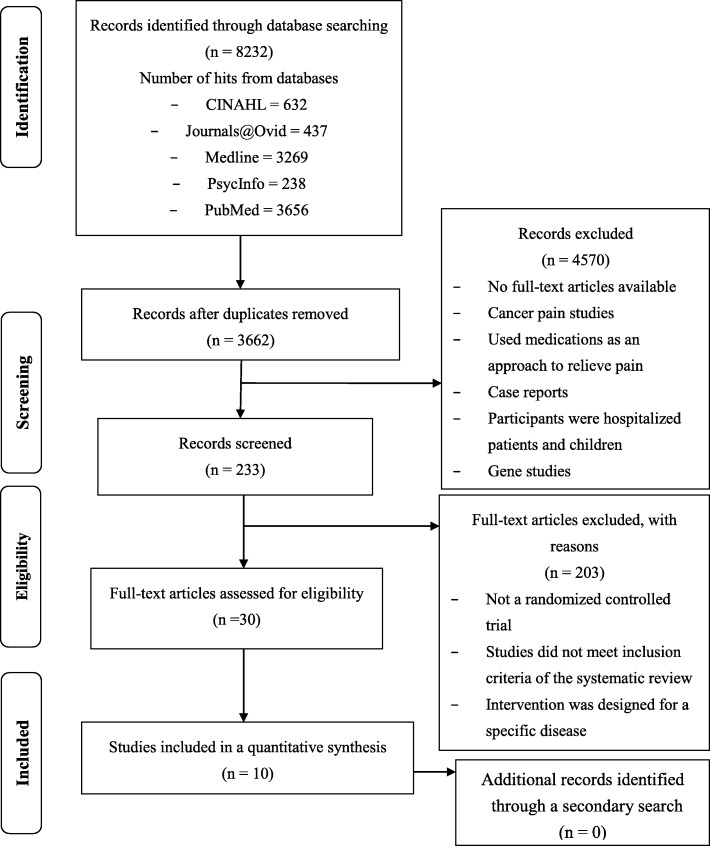
Flow diagram of the study selection process

The initial search of the five internet-based databases yielded 8232 articles. After the removal of duplicates, 3662 articles remained. The titles and abstracts of the articles were then screened to identify studies related to pain, resulting in 233 articles. Ultimately, we retrieved ten articles that met the criteria for inclusion in this review. The PRISMA checklist (see ‘Additional file 1’) shows the reporting process, and the flow diagram in Fig. 1 illustrates the study selection process.

### Study characteristics

The articles included in the systematic review were categorized by type of intervention. Ten articles were included. There was article on acupressure and one on guided imagery. There were two articles each on acupressure, qigong, periosteal stimulation, and Tai Chi.

The duration of the included studies ranged from 4 weeks to 4 months. Apart from the pre-assessment and immediate post-intervention assessment, most of the studies included a follow-up assessment after the completion of the intervention. The earliest follow-up assessment was found to be 3 weeks after the last intervention. Table 1 shows details of the characteristics of the studies.

**Table 1:** Aims and Study Designs of the Included Articles

Reference	Country	Aims	Duration and intervention	Follow-up assessment
Acupressure				
Li et al., 2018 [35]	United States	To investigate the efficacy of a self-administered acupressure treatment on older adults with knee osteoarthritis	8 weeks 3 visits to the centre in 8 weeks - Acupressure was taught to the participants and applied once daily for 5 days in the study period Weekly phone calls	None
Acupuncture				
Itoh et al., 2006 [36]	Japan	To examine the effectiveness of real acupuncture to trigger points as a treatment for chronic low back pain	12 weeks 2 interventions in 3 weekly sessions, with a washout period of 3 weeks: - Trigger point acupuncture	3 weeks
White et al., 2012 [37]	United Kingdom	To examine if an enhanced nonspecific effect associated with needling is present, to determine the effects of the consultation process and the practitioner, to investigate the efficacy of acupuncture on severe osteoarthritis pain, and to improve interpretation of the quantitative study through a nested qualitative network	8 weeks Intervention twice per week for 4 weeks, with 2 consultations and face-to-face open-ended narrative qualitative interviews as a follow-up assessment	4 to 8 weeks
Guided imagery				
Baird, Murawski, & Wu, 2010 [38]	USA	To investigate the efficacy of guided imagery with relaxation (GIR) on symptom relief and medication use in osteoarthritis patients compared with a sham intervention of planned relaxation	16 weeks 12-min audiotape-guided GIR twice a day	None
Periosteal stimulation (PST)				
Weiner et al., 2007 [39]	USA	To evaluate the efficacy of PST in pain reduction and improved function in older adults with knee osteoarthritis, including those with advanced disease	6 weeks 30-min session once per week for PST	12 weeks
Weiner et al., 2008 [44]	USA	To evaluate the effect of percutaneous electrical nerve stimulation (PENS) with and without general conditioning and aerobic exercise (GCAE) on decreasing pain and enhancing function in older adults with chronic low back pain	6 weeks Twice per week 2 groups: - PENS - PENS and GCAE	24 weeks
Qigong				
von Trott et al., 2009 [40]	Germany	To examine if qigong is more effective than no intervention (waiting list) and exercise therapy in older patients with chronic neck pain	12 weeks 24 sessions (45 min each), 2 sessions per week: - Qigong - Exercise therapy	24 weeks
Yang et al., 2005 [41]	Korea	To evaluate the short-term and residual effects of Qi-therapy on chronic pain and mood in older adults	4 weeks 20 min per session, twice per week: External Korean Qi-therapy	2 weeks
Tai chi				
Brismee et al., 2007 [42]	USA	To examine the effects of group and home video Tai Chi exercise intervention programmes To evaluate the sustainability of the effects of Tai Chi on osteoarthritis after cessation of the exercise intervention To incorporate measurements taken at intermediate time points to evaluate and compare them with the pre- and post-measurements in previous studies To apply a standardized form of Tai Chi exercise that has been most widely used in published longitudinal Tai Chi studies for various health conditions	12 weeks Tai Chi exercise programme (24-form simplified Yang-style Tai Chi), 6-week group Tai Chi, 3 40-min sessions per week, another 6-week home Tai Chi programme	6 weeks
Fransen et al., 2007 [43]	Australia	To test whether hydrotherapy or Tai Chi classes are accepted by patients with chronic symptomatic osteoarthritis of the hips or knees as physical activity options that can provide measurable improvements in joint pain and physical function	12 weeks 1-h class twice a week: - Hydrotherapy programme - Tai Chi: modification of 24 forms from the Sun style of Tai Chi	24 weeks

### Quality appraisal

All of the studies described themselves as randomized controlled trials and seven articles presented their methods of randomization. Only Itoh et al.’s study of acupuncture and Li et al.’s study of acupressure were described as double-blinded studies and included details of the method of double blinding [35, 36]. Seven researchers gave details of the withdrawals and dropouts, including the reasons for them. The studies generated scores ranging from two to five out of five points. Table 2 shows the results of the quality appraisal.

**Table 2:** Quality Appraisal of the Included Studies

Reference	Described as randomized	Appropriate randomization	Described as double blinded	Appropriate double blinding method	Described withdrawals and dropouts	Total (5 points)
Acupressure						
Li et al., 2018 [35]	✓	✓	✓	✓	✓	5
Acupuncture						
Itoh et al., 2006 [36]	✓	✓	✓	✓	✓	5
White et al., 2012 [37]	✓	✓	✗	NA	✓	3
Guided imagery						
Baird, Murawski, & Wu, 2010 [38]	✓	✓	✗	NA	✗	2
Periosteal stimulation (PST)						
Weiner et al., 2007 [39]	✓	✗	✗	NA	✓	2
Weiner et al., 2008 [44]	✓	✓	✗	NA	✓	3
Qigong						
von Trott et al., 2009 [40]	✓	✓	✗	NA	✗	2
Yang et al., 2005 [41]	✓	✗	✗	NA	✗	
Tai chi						
Brismee et al., 2007 [42]	✓	✓	✗	NA	✓	3
Fransen et al., 2007 [43]	✓	✓	✗	NA	✓	3

### Results of the included studies

#### Demographics of the participants and pain assessment tools

There were no significant differences in the demographic data of the intervention and control groups in any of the included studies. The number of recruited participants ranged from 26 to 221. The mean age of the participants ranged from 66.75 to 76 years. Three studies did not record the use of medications by the participants [35, 41, 43]. The studies adopted different pain rating scales. Commonly used scales included the visual analogue scale, numeric rating scales, and the Western Ontario and McMaster University Osteoarthritis Index (WOMAC).

#### Non-pharmacological interventions that were included and their effectiveness

All of the studies demonstrated the effectiveness of non-pharmacological interventions for reducing pain in community-dwelling older adults, as demonstrated by decreases in pain intensity from a comparison of the baseline and post-intervention data. Pain intensity data were converted into numeric scores for comparisons between studies, and are shown in Additional file 2: Table S1. The studies covered the interventions of acupressure, acupuncture, guided imagery, periosteal stimulation, qigong, and Tai Chi [35–44]. The net change in pain intensity in the intervention group in the post-intervention assessment ranged from − 3.13 to − 0.65 after the conversion. However, although improvements in pain intensity were found, statistically significant reductions in pain intensity were not found in all of the studies. No statistically significant reductions in pain intensity were found in the studies of White et al., Baird et al., Weiner et al., and von Trott et al. [37–40]. The studies of Li et al., Itoh et al., Yang et al., Brismee et al., Fransen et al., and Weiner et al. reported a statistically significant reduction of *p* < .01 or *p* < .05 [35, 36, 41–44].

#### Suitability and sustainability of the included studies

After the study period, the older adults could continue to implement some of the non-pharmacological interventions. In the study, the older adults were taught to perform the following interventions by themselves: acupressure, guided imaginary, qigong, and Tai Chi [35, 38, 40–43]. Acupuncture and periosteal stimulation required a therapist to perform the intervention. With regard to the sustainability of the interventions, statistically significant decreases in pain intensity in the follow-up assessment were demonstrated in the studies on acupuncture, periosteal stimulation, qigong, and Tai Chi [36, 39–44]. No follow-up assessment was conducted in the studies on acupressure, acupuncture, and guided imagery [35, 37, 38]. Details of the results are presented in Additional file 2: Table S1.

## Discussion

### Summary of evidence

#### Effectiveness of non-pharmacological pain management in community-dwelling older adults

The included non-pharmacological interventions worked well in the older adults in that they had an immediate pain-relieving effect. The pain intensities of the participants in the intervention groups decreased significantly in most of the interventions, for example, the acupressure, qigong, Tai Chi, and hydrotherapy interventions [35, 40, 43]. The other non-pharmacological interventions led to a reduction in pain intensity in the older adults, although without statistical significance. The older adults benefitted directly from lower pain intensity and possibly from a better quality of life, such as through enhancements in mobility and in their ability to carry out the activities of daily living. However, no conclusion can be drawn about which interventions offer the best pain reduction effect in community-dwelling older adults. Further research to compare interventions is recommended to determine the duration of the reduction in pain and the best interventions for decreasing pain in community-dwelling older adults, and to explore the clinical significance of the non-pharmacological interventions.

#### Suitability of non-pharmacological pain management in community-dwelling older adults

It is essential to equip community-dwelling older adults with adequate self-help skills and techniques to manage chronic disease. As pain is present in 25 to 50% of community-dwelling older adults, providing them pain with management skills may be one of the solutions to promoting better health and quality of life [2].

Some of the included articles indicated that certain interventions can be continuously implemented by the older adults themselves. These interventions were acupressure, guided imagery, qigong, and Tai Chi. Satisfactory reductions in pain intensity immediately after the interventions were shown in these studies [35, 38, 40–43]. The studies showed that the pain reduction effects were maintained in the follow-up assessment, with participant dropout rates of 3 to 24% [40–43]. The older adults were able to practise the interventions when they were in pain. The interventions became treatment options that were available to them at all times, and they had the ability to perform these interventions themselves. This is in alignment with the concept of self-management held by the older adults.

#### Sustainability of non-pharmacological pain management in community-dwelling older adults

The sustainability of the interventions refers to whether the participants can self-administer the interventions and maintain the pain reduction effect after the study period. Acupuncture and periosteal stimulation resulted in significant reductions in pain [36, 37, 39, 44]. However, the highest level of decrease in pain was sustained only immediately or for a short time during the post-intervention period. In other words, the interventions need to be delivered on a regular basis to promote the sustainability of the pain reduction. Also, older adults cannot implement these interventions by themselves because an acupuncturist is required to perform them. As a result, older adults can only learn about the concepts and benefits of the interventions, but cannot implement the interventions by themselves whenever they are in pain. Thus, the application of these particular interventions is limited. Another concern is whether the older adults have the ability to continue to self-apply the non-pharmacological interventions. Even though the interventions are suitable for older adults and they were taught the relevant self-application method, their cognitive function and ability to continue using the method remains in question. Their ability to follow the application guidelines and the dosage of the intervention may need to be monitored by a nurse or caregiver. Therefore, it is recommended that the caregiver of an older adult, such as a family member, learn and perform the intervention in order to ensure its effectiveness and safety.

The immediate and short-term effects of pain reduction were shown in the included non-pharmacological pain management studies. However, further investigations are needed to determine the long-term effectiveness of the interventions in managing pain.

### Strengths

The review identified effective non-pharmacological interventions for managing chronic pain that were suitable and sustainable for community-dwelling older adults. Articles from the most recent 10 years were retrieved. PRISMA was adopted to guide the systematic review process. The systematic review provides ideas to nurses about the use of non-pharmacological methods of managing pain in older adults.

### Limitations

The present systematic review has some limitations. A limited number of relevant articles were found in the review. It is possible that the combinations of search terms used resulted in inadequate coverage of the relevant articles. Also, the aim of the systematic review was to examine the effectiveness of non-pharmacological interventions for managing pain. Diverse interventions were included, and there was no in-depth investigation of individual interventions. In addition, no measurement was used to assess the suitability of the interventions used by the older adults. Only articles with pain intensity as the primary outcome were included. It is likely that the review failed to identify studies measuring pain as a secondary outcome in which pain may also have been reduced. The pain intensities in the studies were not standardized. Community-dwelling older adults comprised the population in the systematic review, and the results cannot be generalized to other populations. Only articles in the electronic databases that were published in English were included in the search. Therefore, the analysis in the review may be incomplete, as articles published in other languages have not been studied.

## Conclusions

Ten articles were included in the systematic review. Acupressure, acupuncture, guided imagery, qigong, periosteal stimulation, and Tai Chi were identified as non-pharmacological interventions that provided adequate pain management for community-dwelling older adults, were suitable for them to use, and had sustainable pain reduction effects. Effectiveness, suitability and sustainability should be elements that researchers take into consideration when they design a non-pharmacological intervention for pain management. These definitely can help to further reduce the intensity of the pain felt by older adults and improve their health, making it possible for them to stay in the community. One concern, however, was how to maximize the effects of pain management and maintain the sustainability of the pain reductions. In planning future studies on this topic, we suggest that researchers focus on equipping community-dwelling older adults with the skills that they need to improve their self-efficacy in managing pain, taking into consideration their cognitive function and ability to perform the non-pharmacological pain management interventions, rather than solely investigating the effectiveness of such interventions.

## Supplementary information

**Additional file 1.** PRISMA 2009 checklist. This was the PRISMA checklist used to report the systematic review.

**Additional file 2: Table S1.** Results of the Included Studies.

## Data Availability

The datasets used and/or analysed during this study are available from the corresponding author on reasonable request.

## Abbreviations

MeSH: Medical Subject Headings
MPQ: McGill Pain Questionnaire
NRS: Numeric rating scale
PRISMA: Preferred Reporting Items for Systematic Reviews and Meta-Analyses
SF-MPQ: The short-form McGill Pain Questionnaire
VAS: Visual analogue scale
WOMAC: Western Ontario and McMaster Universities osteoarthritis index
