# Supplementary material for: The effectiveness, suitability, and sustainability of non-pharmacological methods of managing pain in community-dwelling older adults: a systematic review
Source: BMC Public Health. 2019 Nov 8;19:1488. doi: 10.1186/s12889-019-7831-9 (PMC6842175; doi:10.1186/s12889-019-7831-9)
Supplement: Supplementary file 2 — Additional file 2: Table S1. Results of the Included Studies. [file 12889_2019_7831_MOESM2_ESM.docx]

Table S1 *Results of the Included Studies*

| Reference | Recruited N, age, gender | Record of medication use | Pain measurement tool | Results (Converted to a 0 – 10 Numeric Scale) | | | | | | | | | | | | | | | | | | | | | | | | | | | | | | p-value | Pain reduction in follow-up assessment |
| --- | --- | --- | --- | --- | --- | --- | --- | --- | --- | --- | --- | --- | --- | --- | --- | --- | --- | --- | --- | --- | --- | --- | --- | --- | --- | --- | --- | --- | --- | --- | --- | --- | --- | --- | --- |
|  |  |  |  | Intervention group | | | | | | | | | | | | | | | Control group | | | | | | | | | | | | | | |  |  |
|  |  |  |  | Pre | | | | | Post | | | | NC | | | | | | Pre | | | | | | | | Post | | | | NC | | |  |  |
| Acupressure | | | | | | | | | | | | | | | | | | | | | | | | | | | | | | | | | | | |
| Li et al., 2018 | N = 150  Age: 73.5  Females: 93 Males: 57 | No | WOMAC | VA | | | | | | | | | SA | | | | | | | | | | | UC | | | | | | | | | | Estimated *p*: VA-UC: *p* < .001*  SA-UC: *p* < .001*  VA-SA: *p* = .93 | Not available |
|  |  |  |  | Pre | | Post | | | | NC | | | Pre | | | | Post | | | NC | | | | Pre | | | | | Post | | | NC | |  |  |
|  |  |  |  | 3.25 | | 2.4 | | | | -0.85 | | | 3.4 | | | | 2.75 | | | -0.65 | | | | 3.45 | | | | | 3.15 | | | -0.3 | |  |  |
| Acupuncture | | | | | | | | | | | | | | | | | | | | | | | | | | | | | | | | | | | |
| Itoh et al., 2006 | N = 26  Age: 73.5  Females: 17 Males: 9 | Yes | 10-cm VAS | 6.5 | | 4.8 | | | | | | | | | -1.7 | | 6.9 | | | | | | | | 4.38 | | | | | | | -2.52 | | *p* < .01* | Yes |
| White et al., 2012 | N = 221  Age: 66.75  Females: 127 Males: 94 | Yes | 100-mm VAS | RA | |  | | | | | |  | | | SN | |  | | |  | | | | | MES | | | |  | | |  | | RA-SN:  *p* = .40  RA-SE: *p* = .25  SN-SE: *p* = .73 | Not available |
|  |  |  |  | Pre | | Post | | | | | | NC | | | Pre | | Post | | | NC | | | | | Pre | | | | Post | | | NC | |  |  |
|  |  |  |  | 6.05 | | 4.35 | | | | | | -1.7 | | | 5.86 | | 4.4 | | | -1.46 | | | | | 5.83 | | | | 4.92 | | | -0.91 | |  |  |
| Guided imagery | | | | | | | | | | | | | | | | | | | | | | | | | | | | | | | | | | | |
| Baird, Murawski, & Wu, 2010 | N = 30  Age: 70.26  Females: 4 Males: 26 | Yes | 21-point NRS | 4 | | | | | 2.89 | | | | -1.11 | | | | | | 2.74 | | | | | | | | 2.74 | | | | 0 | | | *p* = .284 | Not available |
| Periosteal stimulation | | | | | | | | | | | | | | | | | | | | | | | | | | | | | | | | | | | |
| Weiner et al., 2007 | N = 88  Age: 71.5  Females: 48 Males: 40 | Yes | WOMAC | 9.3 | | | | | 6.17 | | | | -3.13 | | | | | | 9.0 | | | | | | | | 8.04 | | | | -0.96 | | | *p* = .09 | Yes |
| Weiner et al., 2008 | N = 200  Age: PENS 74.1; PENS & GCAE: 73.9  Females: 114 Males: 86 | Yes | Short form of the McGill Pain Questionnaire, pain thermometer | Intervention | | | | | | | | | | | | | | | Control | | | | | | | | | | | | | | | *p*<.01* | Yes |
|  |  |  |  | PENS | | | | | | | | | PENS & GCAE | | | | | | PENS | | | | | | | | | | | PENS & GCAE | | | |  |  |
|  |  |  |  | Pre | Post | | | | NC | | | | Pre | Post | | NC | | | Pre | | | Post | | | | | NC | | | Pre | Post | | NC |  |  |
|  |  |  |  | 2.98 | 2.33 | | | | -0.65 | | | | 2.71 | 1.8 | | -0.91 | | | 2.38 | | | 1.87 | | | | | -0.51 | | | 2.67 | 1.98 | | -0.69 |  |  |
| Qigong | | | | | | | | | | | | | | | | | | | | | | | | | | | | | | | | | | | |
| von Trott et al., 2009 | N = 121  Age: Qigong 75.9; Exercise therapy: 76  Females: 111 Males: 6 | Yes | NPAD | Qigong | | | | | | | | | Exercise therapy | | | | | | | | | | Waiting list control | | | | | | | | | | | *p* = .14 | Yes |
|  |  |  |  | Pre | | | | Post | | | NC | | Pre | | | Post | | | | | NC | | Pre | | | | | Post | | | NC | | |  |  |
|  |  |  |  | 5.64 | | | | 4.74 | | | -0.9 | | 4.71 | | | 4.45 | | | | | -0.26 | | 4.99 | | | | | 5.49 | | | +0.5 | | |  |  |
| Yang et al., 2005 | N = 43  Age: 72.58  Females: 32 Males: 8 | No | 0-100 VAS | 8.0 | | | | | 5.0 | | | | -3.0 | | | | | | 7.5 | | | | | | | 9.0 | | | | | 2.5 | | | *p*<.001* | Yes |
| Tai chi | | | | | | | | | | | | | | | | | | | | | | | | | | | | | | | | | | | |
| Brismee et al., 2007 | N = 41  Age: 70.8  Females: 32 Males: 8 | Yes | 10-cm VAS | 5.66 | | | | | 3.3 | | | | -2.36 | | | | | | 5.41 | | | | 4.63 | | | | | | | | -0.78 | | | *p*<.05** | Yes |
| Fransen et al., 2007 | N = 141  Age: 70.8  Females: 112 Males: 29 | No | WOMAC | Tai chi | | | | | | | | | Hydrotherapy | | | | | | | | | | Waiting list control | | | | | | | | | | | *p* = .002* | Yes |
|  |  |  |  | Pre | | | Post | | | NC | | | Pre | | | | | Post | | NC | | | Pre | | | | | Post | | | NC | | |  |  |
|  |  |  |  | 4.03 | | | 3.07 | | | -0.96 | | | 3.82 | | | | | 2.73 | | -1.09 | | | 4.44 | | | | | 4.04 | | | -0.4 | | |  |  |

*Note.* **p* < .01 and ***p* < .05 are considered to denote statistical significance. NC = net change; VAS = visual analogue scale; VA = Verum acupressure; SA = Sham acupressure; UC = Usual care; RA = real acupuncture; SN = Streitberger needle; MES = mock electrical stimulation; SE: Sham electrical; NRS = numeric rating scale; WOMAC = Western Ontario and McMaster Universities Osteoarthritis Index; PENS = percutaneous electrical nerve stimulation; GCAE = general conditioning and aerobic exercise; NPAD = Neck Pain and Disability Scale.
